# Supplementary material for: Association of Atherogenic Index of Plasma with Cardiometabolic Risk Factors and Markers in Lean 14-to-20-Year-Old Individuals: A Cross-Sectional Study
Source: Children (Basel). 2023 Jun 30;10(7):1144. doi: 10.3390/children10071144 (PMC10378605; doi:10.3390/children10071144)
Supplement: Supplementary file 1 [file children-10-01144-s001.zip › children-2481329-supplementary.pdf]

children

MDPI

Supplementary Materials

Association of Atherogenic Index of Plasma with Cardiometabolic Risk Factors and Markers in Lean 14-to-20-Year-Old Individuals: A Cross-Sectional Study

Katarína Šebeková [1,*], Radana Gurecká [1,2], Melinda Csongová [1], Ivana Koborová [1] and Peter Celec [1,3]

1 Institute of Molecular BioMedicine, Faculty of Medicine, Comenius University, 81108 Bratislava, Slovakia
2 Institute of Medical Physics, Biophysics, Informatics and Telemedicine, Faculty of Medicine, Comenius University, 81108 Bratislava, Slovakia
3 Institute of Pathophysiology, Faculty of Medicine, Comenius University, 81108 Bratislava, Slovakia
* Correspondence: katarina.sebekova@imbm.sk; Tel.: +421-2-9011-9-429

Among 1285 males, 109 (8.5%) presented with AIP≥0.11. Of 109 males with increased atherogenic risk, 72 (66.1%) were overweight/obese. The prevalence of AIP≥0.11 among 373 males with overweight/obesity reached 19.3%.

Out of 1374 females, 48 (3.6%) displayed AIP≥0.11. Among those with increased atherogenic risk, 20 were overweight/obese. The prevalence of AIP≥0.11 among 274 overweight/obese females reached 7.3%.

Supplementary Table S1: Data according to the quartiles of atherogenic index of plasma (AIP) in males.

	Q1 (n=218)<br>-0.90 - ≤-0.37	Q2 (n=219)<br>>-0.37 - ≤-0.25	Q3 (n=219)<br>>-0.25 - ≤-0.12	Q4 (n=219)<br>>-0.12 - <0.11	p
AIP	-0.47±0.08	-0.31±0.04***	-0.19±0.04$^{***,+++}$	-0.03±0.06$^{***,+++,\#\#\#}$	**<0.001**
Age, years	17.0±1.5	17.1±1.3	17.4±1.4	17.3±1.3	0.072
SBP, mm Hg	120±11	120±11	120±12	121±12	0.893
DBP, mm Hg	71±7	72±7	72±7	72±8	0.300
HR, b/min	76±12	78±11	77±15	78±13	0.386
FPG, mmol/l	4.9±0.4	4.9±0.4	4.9±0.4	4.9±0.4	0.175
Uric acid, µmol/l	340±59	343±57	344±56	349±56	0.464
eGFR, ml/s/1.73m^2	112±21	112±21	111±21	108±21	0.153
Hcy, µmol/l	10.8 (7.2; 16.2)	11.0 (7.8; 15.4)	11.5 (8.0; 16.7)	11.4 (7.9; 16.5)	0.194
**Prevalence**					**p_{chi}**
SBP≥130 mm Hg, n (%)	44 (20.2)	33 (15.1)	41 (18.7)	45 (20.5)	0.438
DBP≥85 mm Hg, n (%)	6 (2.8)	8 (3.7)	11 (5.0)	15 (6.8)	0.188
Elevated SBP or DBP, n (%)	45 (20.6)	36 (16.4)	43 (19.6)	53 (24.2)	0.244
Glucose≥5.6 mmol/l, n (%)	12 (5.5)	18 (8.2)	11 (5.0)	15 (6.8)	0.519

Q quartile; SBP systolic blood pressure; DBP diastolic blood pressure; HR heart rate; FPG fasting plasma glucose; eGFR estimated glomerular filtration rate; Hcy homocysteine; Chi chi-square; data are given as mean ± SD (normally distributed data) or as back-transformed log geometric mean (-1SD; +1SD) for data not fitting to normal distribution; data were compared using the ANOVA test with post-hoc Bonferroni correction.

Supplementary Table S2: Data according to the quartiles of atherogenic index of plasma (AIP) in females.

	Q1 (n=268)<br>-1.02 - ≤-0.42	Q2 (n=268)<br>>-0.42 - ≤-0.29	Q3 (n=268)<br>>-0.29 - ≤-0.17	Q4 (n=268)<br>>-0.17 - <0.11	p
AIP	-0.53±0.09	-0.38±0.04***	-0.23±0.03$^{***,+++}$	-0.07±0.07$^{***,+++,\#\#\#}$	**<0.001**
Age, years	17.1±1.4	17.2±1.4	17.2±1.4	17.4±1.3	0.226
SBP, mm Hg	106±9	106±9	106±9	107±9	0.424
DBP, mm Hg	69±8	69±7	70±7	70±8	0.433
HR, b/min	81±13	81±13	80±13	82±11	0.516
FPG, mmol/l	4.7±0.04	4.7±0.04	4.6±0.04	4.7±0.04	0.434
Uric acid, µmol/l	251±50	253±49	255±49	252±48	0.852
eGFR, ml/min/1.73m^2	108±16	108±16	106±16	107±16	0.515
Hcy, µmol/l	9.1 (6.9; 12.0)	9.4 (7.0; 12.7)	9.5 (6.8; 13.3)	9.6 (7.2; 12.8)	0.189
**Prevalence**					**p_{chi}**
SBP≥130 mm Hg, n (%)	6 (2.2)	2 (0.7)	2 (0.7)	2 (0.7)	0.257
DBP≥85 mm Hg, n (%)	11 (4.1)	7 (2.6)	8 (3.0)	8 (3.0)	0.779
Elevated SBP or DBP, n (%)	14 (5.2)	9 (3.4)	9 (3.4)	9 (3.4)	0.593
Glucose≥5.6 mmol/l, n (%)	4 (1.5)	6 (2.2)	3 (1.1)	4 (1.5)	0.768

Q quartile; SBP systolic blood pressure; DBP diastolic blood pressure; HR heart rate; FPG fasting plasma glucose; eGFR estimated glomerular filtration rate; Hcy homocysteine; Chi chi-square; data are given as mean ± SD (normally distributed data) or as back-transformed log geometric mean (-1SD; +1SD) for data not fitting to normal distribution; data were compared using the ANOVA test with post-hoc Bonferroni correction.

Supplementary Table S3: Correlations between cardiometabolic factors and markers and atherogenic index of plasma (AIP) in lean males and females.

	All					Low risk				
	Males (n=912)		Females (n=1100)		P r to z	Males (n=875)		Females (n=1072)		P r to z
	r	p	r	p		r	p	r	p	
SBP	0.026	0.435	0.033	0.277	0.873	0.025	0.456	0.032	0.292	0.881
Heart rate	0.022	0.508	0.056	0.065	0.447	0.048	0.156	0.056	0.066	0.857
FPG	0.043	0.193	-0.029	0.340	0.757	0.049	0.150	-0.033	0.287	0.072
Uric acid	0.056	0.093	0.004	0.889	0.246	0.053	0.118	0.008	0.788	0.322

SBP systolic blood pressure; FPG fasting plasma glucose; r Pearson correlation coefficients; Fisher´s r to z transformation was used to assess the significance of the difference between two correlation coefficients in two independent samples (males vs. females).
